# Biomarkers mining for spinal cord injury based on integrated multi-transcriptome expression profile data

**DOI:** 10.1186/s13018-021-02392-8

**Published:** 2021-04-16

**Authors:** Chongcheng Gong, Lin Liu, Yang Shen

**Affiliations:** Emergency Trauma Surgery, Shanghai East Hospital of Tongji University, No. 150, Jimo Road, Shanghai, 200120 China

**Keywords:** Spinal cord injury, lncRNA, miRNA, mRNA, Competing endogenous RNA

## Abstract

**Background:**

This study was aimed to discover more biomarkers associated with spinal cord injury (SCI) by constructing a competing endogenous RNA (ceRNA) network.

**Methods:**

The transcriptome expression profile data related to SCI (GSE45006 GSE20907) were downloaded from GEO database. The differentially expressed RNAs (DERs), including lncRNAs, miRNAs, and mRNAs, between SCI and control groups were selected, which were then performed function enrichment analyses. Following that, a SCI-related ceRNA regulatory network was constructed. PCA analysis was performed on the genes constituting the ceRNA regulatory network directly related to SCI.

**Results:**

In GSE45006 and GSE20907 datasets, there were respectively 3336 and 1453 DERs. Venn analysis showed that there were 429 DERs which had consistent differential expression direction. RGD1564534-miR-29b-5p relation pair and 103 miRNA-target regulatory pairs were integrated to construct the ceRNA regulatory network. Then a SCI-related ceRNA regulatory network including 8 mRNAs of *IFNGR1*, *STAT2*, *CYBB*, *NFATC1*, *FCGR2B*, *HMOX1*, *TLR4*, and *HK2*, a lncRNA of RGD1564534, and a miRNA of miR-29b-5p was constructed. Additionally, two pathways, osteoclast differentiation, and HIF-1 signaling pathway, were involved in this network. PCA indicated the samples before and after injury can be significantly distinguished based on the genes in the ceRNA network.

**Conclusion:**

A total of 8 SCI-related mRNAs have been identified in the ceRNA network, including *IFNGR1*, *STAT2*, *CYBB*, *NFATC1*, *FCGR2B*, *HMOX1*, *TLR4*, and *HK2.* Moreover, RGD1564534 may serve as ceRNA by competitively binding to miR-29b-5p to regulate the expression of 8 SCI-related mRNAs. Therefore, these genes may serve as key biomarkers of SCI.

## Introduction

Spinal cord injury (SCI) is a disabling disorder, which often results in neurological impairments for the patients with it [1]. Among the patients with SCI, 80% of them experience a severe sensory deficit-neuropathic pain [2]. Moreover, SCI can also lead to pathological changes in the urinary and gastrointestinal systems, increased pulmonary complications, muscle atrophy, loss of bone density, and autonomic dysfunction [3–6]. Presently, there is lack of reliable treatment interventions for the patients with severe neurological loss, and most of the medical decisions are to stabilize the patients and prevent further injury [7]. Therefore, it is urgent to understand the underlying molecular mechanism of SCI so as to provide useful clues for its treatment.

Translational medicine is a kind of new information, new mechanisms, and new technologies that are created by the results of basic science research and effectively converted into new methods for the prevention, diagnosis, and treatment of diseases that are necessary for improving health [8]. Translational research is mainly divided into two sections. One is to transform the new understanding of disease mechanisms obtained in the laboratory into new methods of diagnosis, treatment, and prevention; the other is to transform clinical research results into daily clinical practice and health decision-making [9]. At present, large-scale SCI experiments take many years to complete, while many smaller clinical trials have been halted and most of them have not yet been completed [10]. Therefore, it is important to discover more biomarkers related to SCI by constructing a network of competitive endogenous RNA (ceRNA). Study has reported that the pathophysiological events during SCI are regulated by several specific genetic entities [11, 12], which include both protein-coding messenger RNAs (mRNAs) and non-coding RNAs (ncRNAs). Among the ncRNAs, the sequences of long non-coding RNAs (lncRNAs) are the least evolutionarily conserved. Previous studies have demonstrated the critical roles of lncRNAs, like lncRNA-XIST, and MALAT1, in the pathogenesis of SCI [13, 14]. Importantly, some recent study has indicated that lncRNAs can act as ceRNAs to regulate the expression of mRNAs by competitively binding to microRNAs (miRNAs) [15]. Recently, Wang et al. [16] performed ceRNA network analysis in SCI and found that lncRNA XR_350851 was closely associated with autophagy. Nevertheless, in order to further elucidate the biological processes underlying different stages of SCI, the expression patterns of these molecules need to be clearly investigated.

In this study, the expression levels of transcriptomes in spinal tissue of rats with different stages of SCI were integrated to study the changes of transcriptomes before and after SCI by constructing a ceRNA network, so as to explore the potential mechanisms of SCI. Our results may provide theoretical basis for the treatment of SCI.

## Materials and methods

### Data collection and preprocessing

The transcriptome expression profile data related to SCI were searched in the NCBI GEO [17] in the database, with the keywords of “spinal cord injury.” The criteria for screening the datasets were as follows: (1) the dataset must be the same species (human, rat, mouse, etc.); (2) the tissues used for study must be uniform tissues (spinal column, blood, spinal fluid, etc.); (3) the data set must had before-and-after damage controls, and each state samples must have duplicates. After screening, two datasets meeting the requirements were included in the analysis: GSE45006 (GPL1355 [Rat230_2] Affymetrix Rat Genome 230 2.0 Array) and GSE20907 (GPL6247 [Ragene-1_0-ST] Affymetrix Rat Gene 1.0 ST Array [transcript (Gene) version]). GSE45006 contains four spinal tissue samples of 2 weeks after injury and 4 control samples, and GSE20907 contains two spinal tissue samples of 2 weeks after injury and 4 control samples.

We downloaded the original data with format of CEL, and performed original CEL format data conversion, missing value supplement (median method), and data normalization (MAS method and quantiles method) using the R3.6.1 oligo version 1.41.1 [18].

The detail platform annotation information, including probe, gene symbol, and RNA types, was downloaded from Ensembl genome browser 96, and then the detection probes downloaded from the two datasets were reannotated to obtain the expression levels corresponding to lncRNA, miRNA, and mRNA.

### Differentially expressed RNA selection

In the two datasets, Limma version 3.34.0 [19] was used to screen significantly differentially expressed RNAs (DERs) with thresholds of false discovery rate (FDR) < 0.05 and |log2 fold change (FC)| > 0.263. Then based on the obtained DERs, bidirectional hierarchical clustering was performed for the expression values using pheatmap package (version 1.0.8) [20].

Then by comparing the DERs from GSE45006 and GSE20907 datasets, the intersection DERs with the same expression direction were considered as the common DERs of the two datasets. These DERs were subjected to GO biological processes and KEGG pathway enrichment analyses using DAVID version 6.8 [21, 22] and *p* value < 0.05 was used as the threshold.

### Construction of ceRNA regulatory network

We first downloaded the sequences of target lncRNAs from Ensembl Genome Browser96 database, and downloaded the sequences of target miRNAs from miRBase. Then the binding relationship between target lncRNAs and miRNAs was predicted through miRanda [23] (miRanda comparison parameters: Gap Open Penalty = −8, Gap Extend = −2, Score Threshold = 80%, Energy Threshold = −20). The lncRNAs and miRNAs with opposite difference direction were reserved.

The target genes regulated by the target miRNAs were searched in TargetScan [24, 25]. Then the obtained common DE mRNAs were mapped to the target gene to build the miRNA-mRNA regulatory relations.

Finally, the lncRNA-miRNA and miRNA-mRNAs were synthesized, and a ceRNA regulatory network composed of lncRNA-miRNA-mRNAs was constructed, which was demonstrated by Cytoscape 3.6.1 [26]. Then, the target genes in the ceRNA regulatory network were annotated with GO biological process and KEGG pathway using DAVID 6.8 [21, 22].

### Construction of ceRNA regulatory network directly related to SCI

Based on “spinal cord injury” as the keyword, KEGG pathways directly related to SCI were searched in the Comparative Toxicogenomics Database 2019 update [27]. These pathways were then compared with the KEGG pathways obtained from the ceRNA network above to identify the overlapped pathways. The ceRNA regulatory network related to the genes involved in the overlapped pathways was extracted separately, which was considered as the SCI pathway-related ceRNA regulatory network.

### Principal component analysis of SCI pathway genes

Principal component analysis (PCA) is a dimension reduction technique, which can transform multiple variables into a few principal components that can reflect most of the information of the original variable [28]. Using R3.6.1 psych package version 1.7.8, PCA analysis was performed on the genes constituting the ceRNA regulatory network directly related to SCI to observe the differentiation of genes involved in SCI pathway in different types of samples and the principal component relationship of expression level.

## Results

### DERs identification

**Fig. 1:**
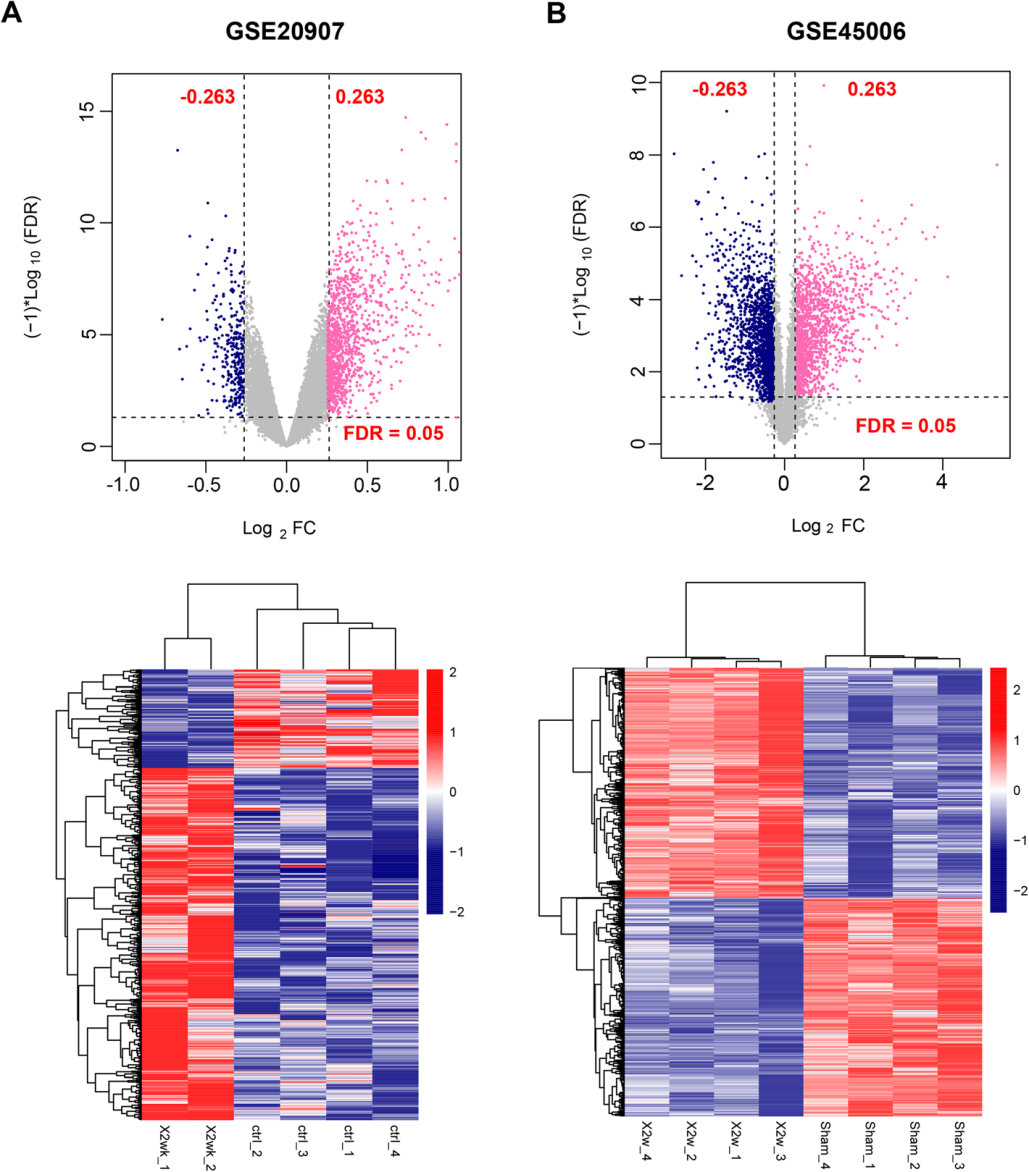
Volcano plot and heatmaps for differentially expressed RNAs in GSE20907 (**a**) and GSE45006 (**b**)

In GSE45006 and GSE20907 datasets, there were respectively 3336 and 1453 DERs. The volcano plots and heatmaps are shown in Fig. 1. In both datasets, samples belonging to the same category tended to cluster together. Venn analysis showed that there were 535 overlapped DERs between two datasets, of which 429 had consistent differential expression direction, including 1 lncRNA, 2 miRNAs, and 426 mRNAs.

### Function enrichment analysis

**Fig. 2:**
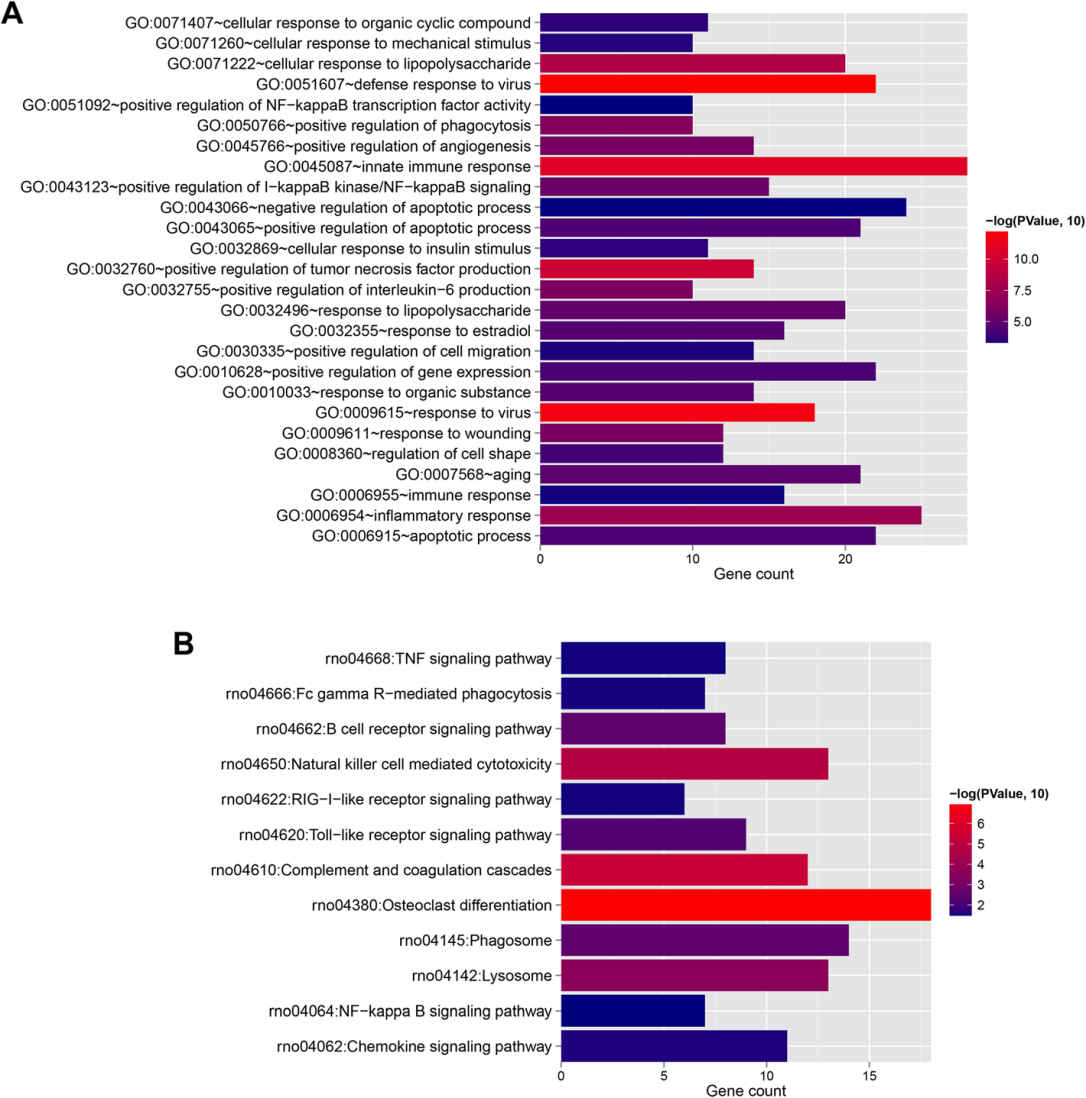
Histogram of GO biological process (**a**) and KEGG signaling pathway (**b**) with significant correlation with differentially expressed mRNAs. Horizontal axis represents the number of genes, vertical axis represents the item name, point size represents the number of genes, and color represents the significance

The 426 mRNAs were performed GO and KEGG analyses, and 26 GO biological processes (Fig. 2a) and 12 pathways (Fig. 2b) were identified. The top 5 GO terms with low *p* values included GO:0051607~defense response to virus, GO:0009615~response to virus, GO:0045087~innate immune response, GO:0032760~positive regulation of tumor necrosis factor production, GO:0071222~cellular response to lipopolysaccharide, GO:0006954~inflammatory response, GO:0050766~positive regulation of phagocytosis, GO:0009611~response to wounding, GO:0032755~positive regulation of interleukin-6 production, and GO:0045766~positive regulation of angiogenesis. The top 5 pathways with low *p* values were rno04380: osteoclast differentiation, rno04610: complement and coagulation cascades, rno04650: natural killer cell mediated cytotoxicity, rno04142: lysosome, and rno04145: phagosome.

### ceRNA regulatory network construction

**Fig. 3:**
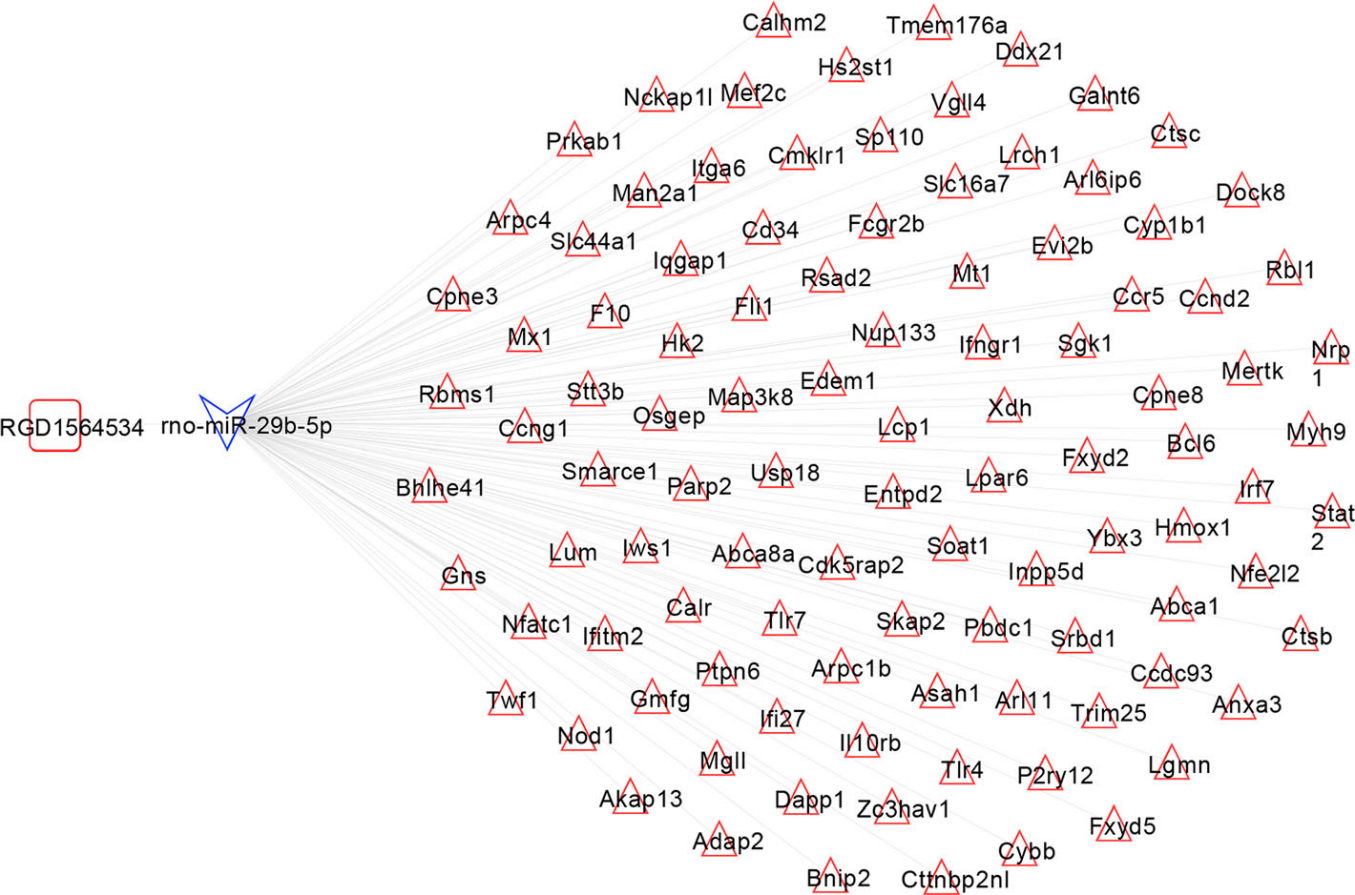
CeRNA regulatory network. The square represents lncRNAs, the triangle represents miRNAs, the triangle represents mRNAs, and the blue and red border colors represent consistent significantly downregulated and upregulated expressions, respectively

The binding relation between 1 lncRNA and 2 miRNAs was predicted. Then combining with the differential expression direction, RGD1564534-miR-29b-5p relation pair was obtained. Following that the target genes of miR-29b-5p was predicted and compared with the DE mRNAs. Among the obtained miRNA-target regulatory pairs, the relation pairs with opposite differential expression directions (a total of 103 pairs) were retained. Finally, the RGD1564534-miR-29b-5p relation pair and 103 miRNA-target regulatory pairs were integrated to construct the ceRNA regulatory network of lncRNA-miRNA-mRNA (Fig. 3).

**Fig. 4:**
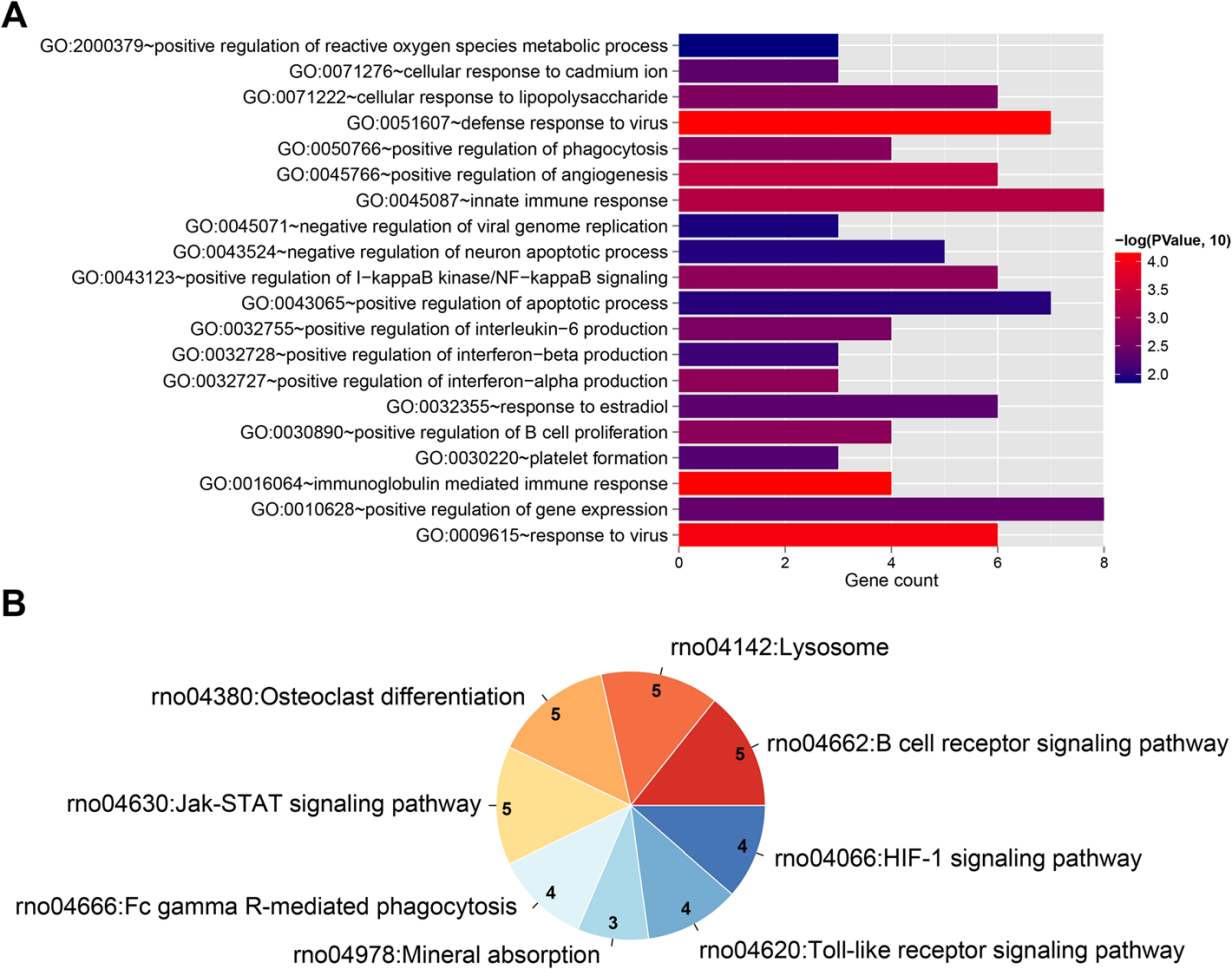
**a** Bar chart of GO biological process with significant correlation with target genes in the network. Horizontal axis represents the number of genes, vertical axis represents the item name, point size represents the number of genes, and color represents the significance. **b** Pie chart of KEGG signaling pathway. The number represents the number of genes involved in the pathway, and the color represents the significance

The mRNAs involved in the ceRNA network were performed function enrichment analysis. A total of 37 GO terms, such as GO:0016064~immunoglobulin mediated immune response, GO: 0051607~defense response to virus, GO: 0009615~response to virus, GO: 0045766~positive regulation of angiogenesis, and GO: 0045087~innate immune response, as well as 8 pathways, such as rno04662: B cell receptor signaling pathway, rno04142: lysosome, rno04380: osteoclast differentiation, rno04630: Jak-STAT signaling pathway, and rno04666: Fc gamma R-mediated phagocytosis were enriched (Fig. 4).

### ceRNA regulatory network directly related to SCI

**Fig. 5:**
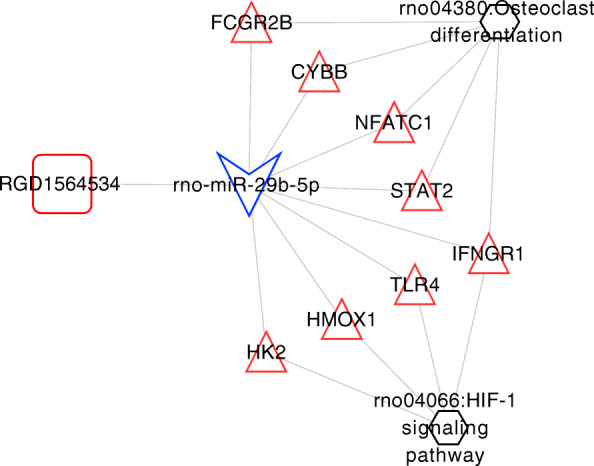
Spinal cord injury pathway-related integrated ceRNA regulatory network. Square represents lncRNAs, triangle represents miRNAs, triangle represents mRNAs, blue and red in border colors represent consistent significantly downregulated and upregulated expression, respectively, and hexagonal nodes represent KEGG signaling pathways

In the CTD database with “spinal cord injury” as keywords, the KEGG pathways directly related to SCI were searched, obtaining 88 KEGG signaling pathways. These pathways were then compared with the 8 pathways obtained from the ceRNA network, and obtained two overlapped pathways: rno04380: osteoclast differentiation, and rno04066: HIF-1 signaling pathway. Then the comprehensive ceRNA regulatory network related to the mRNAs enriched in the two KEGG pathways was separately extracted to construct the SCI-related ceRNA regulatory network, as shown in Fig. 5. This network contained 8 mRNAs of *IFNGR1*, *STAT2*, *CYBB*, *NFATC1*, *FCGR2B*, *HMOX1*, *TLR4*, and *HK2*, a lncRNA of RGD1564534, and a miRNA of miR-29b-5p*.*

### PCA of pathway network elements

In order to further select the important DERs in the network, we used PCA method to screen the important genes with obvious characteristics on expression level. The expression values of 8 mRNAs, 1 lncRNA, and 1 miRNA that constituted the network were extracted from the GSE45006 and GSE20907 datasets. PCA based on gene expression values was performed on the samples to obtain each principal component. The results showed that the cumulative contribution rates of PC1, 2, and 3 exceeded 90%, indicating that the first three principal component factors obtained by fitting contained a lot of information about original variables (RNAs expression values).

**Fig. 6:**
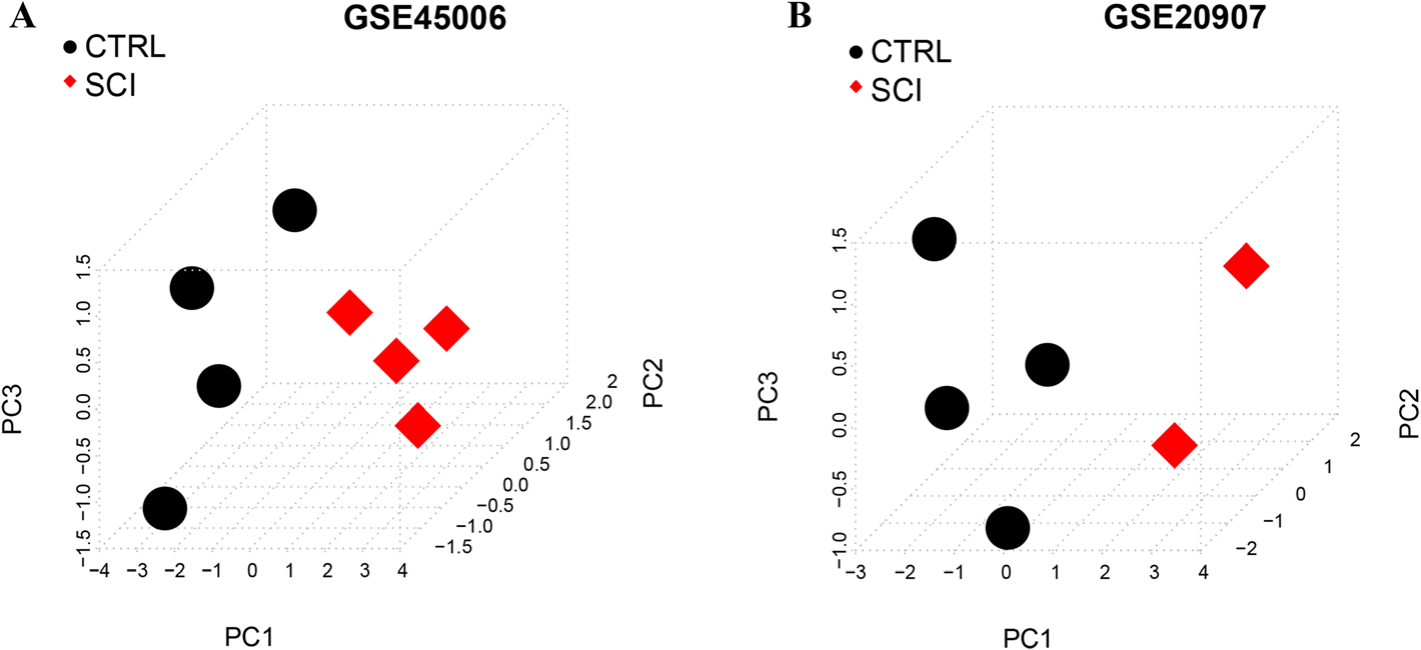
Three-dimensional plots of sample distribution based on PC1, 2, and 3 principal components in GSE45006 (**a**) and GSE20907 (**b**). The black dots represent control samples, and the red dots represent spinal cord injury (SCI) samples

To further verify the characteristics of the first three principal components obtained by fitting in each data set sample, we used the obtained PC1, PC2, and PC3 to make three-dimensional sample graphs, as shown in Fig. 6. Based on the three principal components of PC1, 2, and 3, the samples before and after injury can be significantly distinguished, proving that the first three principal components contained the information of the expression of most of the original variables RNAs.

## Discussion

In the present study, based on the DERs obtained from GSE45006 and GSE20907, a SCI-related ceRNA regulatory network including 8 mRNAs of *IFNGR1*, *STAT2*, *CYBB*, *NFATC1*, *FCGR2B*, *HMOX1*, *TLR4*, and *HK2*, a lncRNA of RGD1564534, and a miRNA of miR-29b-5p was constructed. Additionally, two pathways, rno04380: osteoclast differentiation, and rno04066: HIF-1 signaling pathway, were involved in this network. PCA indicated the samples before and after injury can be significantly distinguished based on the genes in the ceRNA network.

Among the eight mRNAs in the ceRNA network related to SCI, *IFNGR1*, *HMOX1*, *TLR4*, and *HK2* were involved in HIF-1 signaling pathway. Hypoxia-inducible factor 1 (HIF-1) is a major regulator of response to insufficient oxygen supply, which is thought to protect hypoxic cells from apoptosis and necrosis and protect the nervous system from further damage under ischemic and hypoxia conditions [29]. It has been reported that at the early stages of SCI, overexpressed HIF-1 can help induce hypoxia tolerance and regulate the vascularity of the injured spinal cord [30]. Thus, HIF-1 plays an important role in the recovery of SCI [30]. Our results further indicated the critical role of HIF-1 signaling pathway in SCI through regulating *IFNGR1*, *HMOX1*, *TLR4*, and *HK2*.

Osteoclasts are multinucleated cells derived from the fusion of the precursor of hematopoietic osteoclasts, which are responsible for bone resorption and osteoclast differentiation and represent an evident control point of bone resorption [31]. In SCI, bone remodeling becomes uncoupled with an initial reduction in bone formation and a steady increase of bone resorption [32]. Given the role of osteoclasts in bone resorption, we speculated that osteoclasts may have a regulatory effect on SCI progression. In this study, *STAT2*, *CYBB*, *NFATC1*, *FCGR2B*, and *IFNGR1* in the ceRNA network were enriched in the pathway of osteoclast differentiation, which suggested that these genes may be involved in SCI progression via osteoclast differentiation.

There are numerous miRNAs in the central nervous system, which are indispensable for the development of central nervous system [33, 34]. Specially, miRNAs are attractive candidates in SCI. miR-29b has been reported to regulate neuronal apoptosis in brain injuries [35]. Liu et al. [36] recently reported that miR-29b contributed to the neuronal cell death of SCI through upregulating pro-apoptotic BH3-only and downregulating anti-apoptotic myeloid cell leukemia sequence-1 proteins. Additionally, the miR-29b has been reported to support osteoblast differentiation through decreasing the activity of collagen protein, such as collagen type Ι alpha Ι (*COL1A1*), *COL5A3*, and *COL4A2* [37]. As we mentioned above, osteoclast differentiation may be associated with SCI progression; thus, we speculated that miR-29b-5p may serve as a key factor in SCI progression.

There are some limitations to this analysis. First, the study used the probe reannotation method to annotate gene symbol, which could drop some genes because of the failure to match the probes. Second, though this study identified 8 mRNAs, including *IFNGR1*, *STAT2*, *CYBB*, *NFATC1*, *FCGR2B*, *HMOX1*, *TLR4*, and *HK2*, which are significantly related to SCI, further research are still needed to confirm the expression of these genes in SCI. Third, though we identified a ceRNA network, the exact mechanism of this network should be investigated in in vivo and in vitro experiments in future research.

## Conclusion

In conclusion, a total of 429 DERs related to SCI were obtained in this study. A ceRNA network was constructed to analyze the development mechanism of SCI at a system-wide level. A total of 8 SCI-related mRNAs have been identified in the ceRNA network, including *IFNGR1*, *STAT2*, *CYBB*, *NFATC1*, *FCGR2B*, *HMOX1*, *TLR4*, and *HK2*. Moreover, it was also found that RGD1564534 may serve as ceRNA by competitively binding to miR-29b-5p to regulate the expression of those 8 SCI-related mRNAs. Therefore, these genes may serve as key biomarkers of SCI.

## Data Availability

The datasets used and/or analyzed during the current study are available from the corresponding author on reasonable request.

## Abbreviations

SCI: Spinal cord injury
ceRNA: Competing endogenous RNA
DERs: Differentially expressed RNAs
mRNAs: Messenger RNAs
ncRNAs: Non-coding RNAs
lncRNAs: Long non-coding RNAs
miRNAs: MicroRNAs
FDR: False discovery rate
PCA: Principal component analysis

## References

[1] Eckert MJ, Martin MJ (2017). Trauma: spinal cord injury. Surg Clin.

[2] Cragg JJ, Noonan VK, Noreau L, Borisoff JF, Kramer JK (2015). Neuropathic pain, depression, and cardiovascular disease: a national multicenter study. Neuroepidemiology.

[3] Moore C, Craven B, Thabane L, Laing A, Frank-Wilson A, Kontulainen S, Papaioannou A, Adachi J, Giangregorio L (2015). Lower-extremity muscle atrophy and fat infiltration after chronic spinal cord injury. J Musculoskelet Neuronal Interact.

[4] Karapolat I, Karapolat HU, Kirazli Y, Capaci K, Akkoc Y, Kumanlioglu K (2015). Longitudinal study of bone loss in chronic spinal cord injury patients. J Phys Ther Sci.

[5] Ebert E (2012). Gastrointestinal involvement in spinal cord injury: a clinical perspective. J Gastrointest Liver Dis.

[6] Herrera JJ, Haywood-Watson RJ, Grill RJ (2010). Acute and chronic deficits in the urinary bladder after spinal contusion injury in the adult rat. J Neurotrauma.

[7] Albayar AA, Roche A, Swiatkowski P, Antar S, Ouda N, Emara E, Smith DH, Ozturk AK, Awad BI (2019). Biomarkers in spinal cord injury: prognostic insights and future potentials. Front Neurol.

[8] Mediouni M, Schlatterer DR, Madry H, Cucchiarini M, Rai B (2018). A review of translational medicine. The future paradigm: how can we connect the orthopedic dots better?. Curr Med Res Opin.

[9] Rubio DM, Schoenbaum EE, Lee LS, Schteingart DE, Marantz PR, Anderson KE, Platt LD, Baez A, Esposito K (2010). Defining translational research: implications for training. Acad Med.

[10] Elizei SS, Kwon BK (2017). The translational importance of establishing biomarkers of human spinal cord injury. Neural Regen Res.

[11] Finelli MJ, Wong JK, Zou H (2013). Epigenetic regulation of sensory axon regeneration after spinal cord injury. J Neurosci.

[12] Wintzer M, Mladinic M, Lazarevic D, Casseler C, Cattaneo A, Nicholls J (2004). Strategies for identifying genes that play a role in spinal cord regeneration. J Anat.

[13] Gu S, Xie R, Liu X, Shou J, Gu W, Che X (2017). Long coding RNA XIST contributes to neuronal apoptosis through the downregulation of AKT phosphorylation and is negatively regulated by miR-494 in rat spinal cord injury. Int J Mol Sci.

[14] Qiao Y, Peng C, Li J, Wu D, Wang X (2018). LncRNA MALAT1 is neuroprotective in a rat model of spinal cord ischemia-reperfusion injury through miR-204 regulation. Curr Neurovasc Res.

[15] Tay Y, Rinn J, Pandolfi PP (2014). The multilayered complexity of ceRNA crosstalk and competition. Nature.

[16] Wang L, Wang B, Liu J, Quan Z (2019). Construction and analysis of a spinal cord injury competitive endogenous RNA network based on the expression data of long noncoding, micro-and messenger RNAs. Mol Med Rep.

[17] Barrett T, Wilhite SE, Ledoux P, Evangelista C, Kim IF, Tomashevsky M, Marshall KA, Phillippy KH, Sherman PM, Holko M (2012). NCBI GEO: archive for functional genomics data sets—update. Nucleic Acids Res.

[18] Parrish RS, Spencer HJ (2004). Effect of normalization on significance testing for oligonucleotide microarrays. J Biopharm Stat.

[19] Ritchie ME, Phipson B, Wu D, Hu Y, Law CW, Shi W, Smyth GK (2015). Limma powers differential expression analyses for RNA-sequencing and microarray studies. Nucleic Acids Res.

[20] Wang L, Cao C, Ma Q, Zeng Q, Wang H, Cheng Z, Zhu G, Qi J, Ma H, Nian H (2014). RNA-seq analyses of multiple meristems of soybean: novel and alternative transcripts, evolutionary and functional implications. BMC Plant Biol.

[21] Huang D, Sherman BT, Lempicki RA (2009). Systematic and integrative analysis of large gene lists using DAVID bioinformatics resources. Nat Protoc.

[22] Huang DW, Sherman BT, Lempicki RA (2009). Bioinformatics enrichment tools: paths toward the comprehensive functional analysis of large gene lists. Nucleic Acids Res.

[23] Betel D, Koppal A, Agius P, Sander C, Leslie C (2010). Comprehensive modeling of microRNA targets predicts functional non-conserved and non-canonical sites. Genome Biol.

[24] Agarwal V, Bell GW, Nam J-W, Bartel DP (2015). Predicting effective microRNA target sites in mammalian mRNAs. elife.

[25] Chiang HR, Schoenfeld LW, Ruby JG, Auyeung VC, Spies N, Baek D, Johnston WK, Russ C, Luo S, Babiarz JE (2010). Mammalian microRNAs: experimental evaluation of novel and previously annotated genes. Genes Dev.

[26] Shannon P, Markiel A, Ozier O, Baliga NS, Wang JT, Ramage D, Amin N, Schwikowski B, Ideker T (2003). Cytoscape: a software environment for integrated models of biomolecular interaction networks. Genome Res.

[27] Davis AP, Grondin CJ, Johnson RJ, Sciaky D, McMorran R, Wiegers J, Wiegers TC, Mattingly CJ (2019). The comparative toxicogenomics database: update 2019. Nucleic Acids Res.

[28] Alizadeh E, Lyons SM, Castle JM, Prasad A (2016). Measuring systematic changes in invasive cancer cell shape using Zernike moments. Integr Biol.

[29] Umschweif G, Alexandrovich AG, Trembovler V, Horowitz M, Shohami E (2013). Hypoxia-inducible factor 1 is essential for spontaneous recovery from traumatic brain injury and is a key mediator of heat acclimation induced neuroprotection. J Cereb Blood Flow Metab.

[30] Ha X-Q, Yang B, Hou H-J, Cai X-L, Xiong W-Y, Wei X-P (2020). Protective effect of rhodioloside and bone marrow mesenchymal stem cells infected with HIF-1-expressing adenovirus on acute spinal cord injury. Neural Regen Res.

[31] Roux S, Orcel P (2000). Bone loss: factors that regulate osteoclast differentiation-an update. Arthritis Res Ther.

[32] Uebelhart D, Hartmann D, Vuagnat H, Castanier M, Hachen H, Chantraine A (1994). Early modifications of biochemical markers of bone metabolism in spinal cord injury patients. A preliminary study. Scand J Rehabil Med.

[33] Davis TH, Cuellar TL, Koch SM, Barker AJ, Harfe BD, McManus MT, Ullian EM (2008). Conditional loss of Dicer disrupts cellular and tissue morphogenesis in the cortex and hippocampus. J Neurosci.

[34] Tonelli DDP, Pulvers JN, Haffner C, Murchison EP, Hannon GJ (2008). Huttner WB: miRNAs are essential for survival and differentiation of newborn neurons but not for expansion of neural progenitors during early neurogenesis in the mouse embryonic neocortex. Development.

[35] Zhang K, Zhang C, Liu L, Zhou J (2014). A key role of microRNA-29b in suppression of osteosarcoma cell proliferation and migration via modulation of VEGF. Int J Clin Exp Pathol.

[36] Liu X-J, Zheng X-P, Zhang R, Guo Y-L, Wang J-H (2015). Combinatorial effects of miR-20a and miR-29b on neuronal apoptosis induced by spinal cord injury. Int J Clin Exp Pathol.

[37] Zeng Q, Wang Y, Gao J, Yan Z, Li Z, Zou X, Li Y, Wang J, Guo Y (2019). miR-29b-3p regulated osteoblast differentiation via regulating IGF-1 secretion of mechanically stimulated osteocytes. Cell Mol Biol Lett.

